# Host-derived immune signatures as biomarkers to differentiate arboviral acute febrile illness from other causes of acute fever

**DOI:** 10.3389/fmicb.2026.1926383

**Published:** 2026-09-03

**Authors:** Natalia Tiberti, Giulia Bertoli, Cristina Mazzi, Elisabetta Vezzelli, Lorenza Sanfilippo, Matteo Valerio, Leonardo Ancillotti, Silvia Accordini, Chiara Piubelli, Dora Buonfrate, Concetta Castilletti, Federico Giovanni Gobbi

**Affiliations:** 1 Department of Infectious, Tropical Diseases and Microbiology, IRCCS Sacro Cuore Don Calabria Hospital, Negrar di Valpolicella, Italy; 2 Clinical Research Unit, IRCCS Sacro Cuore Don Calabria Hospital, Negrar di Valpolicella, Verona, Italy; 3 Section of Biostatistics, IRCCS Sacro Cuore Don Calabria Hospital, Negrar di Valpolicella, Italy; 4 Infectious and Tropical Diseases Unit, Department of Medical Sciences, University Hospital of Siena, Siena, Italy; 5 Department of Clinical and Experimental Sciences, University of Brescia, Brescia, Italy

**Keywords:** acute febrile illness, acute phase response, arboviruses, biomarkers, chemokines, cytokines

## Abstract

**Introduction:**

Arbovirus infections are emerging or re-emerging global health threats due to their raised incidence and geographical spread in endemic regions, as well as the increased risk of local transmission in non-endemic areas. When symptomatic, arboviral infections usually manifest as acute febrile illnesses (AFI) accompanied by mild, often non-specific symptoms. This non-specific clinical presentation contributes to their misdiagnosis, leading to adverse effects at both the patient and public health levels. Leveraging the potential of host biomarkers as fever triage tools, we assessed their utility for the early identification of arbovirus-associated AFI.

**Methods:**

We retrospectively compared the serum concentrations of 16 immune mediators and 4 haematological parameters (haemoglobin—Hgb, and leukocyte, erythrocyte and platelet counts) in a population of febrile or symptomatic patients with arboviral infections (Arbo-AFI, *n* = 89) or with fever caused by other infectious conditions, including respiratory viral infections (Other-AFI, *n* = 51).

**Results:**

Arbo-AFI was accompanied by increased mediators of the anti-viral response (IFNα, CXCL10, TRAIL and CCL2) and decreased acute phase reactants (CRP, PCT and PTX3) and acute innate cytokines (IL-6 and IL-8) when compared to other causes of AFI. Importantly, some of the best performing markers—including CXCL10, TRAIL, CRP and PCT—showed distinct profiles in Arbo-AFI versus AFI due to respiratory viruses. CRP was the best individual marker (Area Under the ROC Curve—AUC = 0.822), and its diagnostic potential was significantly improved when combined with PCT, CXCL10, IL-6 or Hgb, reaching an AUC > 0.90.

**Discussion:**

We identified novel host-derived immune signatures capable of discriminating acute fever caused by arboviral infections from other infectious aetiologies. Our findings pave the way for prospective validation studies to establish the clinical utility of these markers as triage tools for arboviral fevers.

## 1 Introduction

In recent years, Europe has experienced a rise in both autochthonous (West Nile—WNV, Toscana—TOSV, Tick borne encephalitis—TBEV, Usutu—USUV) and imported (chikungunya—CHIKV, dengue—DENV, Oropouche—OROV, Zika—ZIKV) arboviral infections (Cattaneo et al., 2025; Carbone et al., 2025). Factors contributing to this trend include the geographical expansion of competent vectors, climate change and increased global trade and travels. As a consequence, the risk of local outbreaks triggered by imported viruses has increased, as recently observed with autochthonous transmission events of dengue and chikungunya also in non-endemic areas (Cattaneo et al., 2025; Carbone et al., 2025; Gossner et al., 2018; Buonfrate et al., 2026).

Arboviral infections are often clinically silent. When they cause symptoms, they usually manifest as acute febrile illnesses (AFI) accompanied by mild, often non-specific symptoms, although progression to severe disease can occur (Vellere et al., 2020). This non-specific clinical presentation contributes to the miss-diagnosis of arboviral infections, especially outside specialised referral centres. The timely determination of the arboviral aetiology of AFI is nevertheless paramount to prevent serious untoward effects at the patient and public health levels. AFI of different aetiologies are associated with alterations in circulating immune factors, the most common of which are C reactive protein (CRP), procalcitonin (PCT), leukocyte and neutrophil counts (Leticia Fernandez-Carballo et al., 2021).

In the context of arboviral infections, host-derived factors have been investigated as potential biomarkers for the early detection and assessment of disease severity in DENV, WNV or CHIKV through comparative analyses of infected patients and healthy controls (Patro et al., 2019; Constant et al., 2022; Almeida et al., 2021; Jiravejchakul et al., 2025; Pavesi et al., 2024). Collectively, those investigations suggest that cytokine signatures might characterise the systemic response to these pathogens, with frequent alterations in the levels of IL-6, CCL2 and IL-10, among others. Here, we hypothesised that AFI caused by arboviruses might be accompanied by a specific immune signature that differs from the one induced by other causes of AFI. To address this, we retrospectively compared adult patients with laboratory-confirmed arboviral infections to a heterogeneous group of febrile patients with non-arboviral diagnoses—including bacterial infections, malaria and respiratory viral infections—and assess the potential utility of 16 immune mediators in discriminating arboviral AFI from other causes of fever.

## 2 Materials and methods

Serum samples collected between 2013 and 2025 from febrile (body temperature ≥38 °C) or symptomatic adult patients with a laboratory-based diagnosis of arboviral infections (Arbo-AFI, *n* = 89) or other causes of AFI (Other-AFI, *n* = 51), were included (Supplementary Table S1), based on data retrieved from the Laboratory Information Management System (LIMS) and the corresponding medical records. Arbo-AFI were diagnosed based on positive polymerase chain reaction (PCR) for DENV, ZIKV, WNV, CHIKV, TBEV or OROV in serum, plasma, urine or cerebrospinal fluid (CSF); positive NS1 antigenic test results for DENV; or positive IgM in CSF for TBEV. Each patient was positive to at least one test. Details on the proportion of cases confirmed by each diagnostic method are reported in Supplementary Table S2. Other-AFI encompassed febrile patients with microscopy-based diagnosis of malaria, PCR detection of respiratory viruses (i.e., SARS-CoV-2, Influenza A virus) and clinical and/or laboratory-based diagnosis of bacterial infections. Diagnostic details of the latter group are reported in Supplementary Table S1.

Serum samples were retrospectively selected and retrieved from the Tropica Biobank of the IRCCS Sacro Cuore Don Calabria Hospital (BBMRI-eric ID: IT_1605519998080235). All patients provided written informed consent and the study received ethical approval from the Ethical Committee of Verona and Rovigo Provinces (protocol number 29528, 09/05/2022) and subsequent amendments (Comitato Etico Territoriale Area Sud Ovest Veneto, protocol number 73647, 22/12/2025). The clearance covered the retrospective investigations of samples collected between 2013 and 2025. All sera analysed were leftover of samples collected for diagnostic purposes, thus they were submitted to the same pre-analytical procedures (Tiberti et al., 2025) and measurement performed after only one thawing. Briefly, samples were drawn in vacuum blood collection tubes (CAT Serum Separator Clot Activator Tube, Greiner Bio-One, Kremsmünster, Austria), transported, and stored upright at room temperature until processing. Serum was prepared by centrifugation at 1650 × g for 8 min at room temperature, and then separated into 200–500 μL aliquots in 1.5 mL microtubes (Sarstedt, Nümbrecht, Germany) and stored at −80 °C.

Marker selection was based on literature search considering known pathophysiological features of antiviral and inflammatory pathways (Patro et al., 2019; Constant et al., 2022; Pavesi et al., 2024; Teng et al., 2015; Sivasubramanian et al., 2022; Porte et al., 2019), as well as markers already proposed for the differential diagnosis of acute fever of different aetiologies (Leticia Fernandez-Carballo et al., 2021). Our selection included four haematological parameters, i.e., white blood cell (WBC), erythrocyte (RBC) and platelet (PLT) counts, and haemoglobin (Hgb) levels, and 16 immune mediators, i.e., CCL2, CXCL10, ICAM-1, IFNα, IFNγ, IL-2, IL-4, IL-6, IL-8, IL-10, Procalcitonin (PCT), TNFα, TRAIL, VCAM-1, CRP and Pentraxin 3 (PTX3 or TGS-14). WBC, RBC and PLT counts and Hgb levels were retrieved from electronic medical records. Serum CCL2, CXCL10, ICAM-1, IFNα, IFNγ, IL-2, IL-4, IL-6, IL-8, IL-10, PCT, TNFα, TRAIL and VCAM-1 were determined using a custom Luminex® Discovery assay on a Luminex® 200 analyser and analysed using the Luminex xPONENT® software v4.3. CRP and PTX3 were measured using Simple Plex Human Cartridges on an Ella Instrument (Bio-Techne). All assays were performed following manufacturer’s instructions. To avoid missing values, samples having measured concentration below or above the assay limit of detection were assigned arbitrary values as reported elsewhere (Tiberti et al., 2025).

Differences in marker concentrations between groups were assessed, according to data distribution, through the non-parametric Wilcoxon rank sum test (*p*-value) followed by adjustment for multiple testing using the Benjamini-Hochberg false discovery rate (FDR) method to control for type I error (*q*-values). Markers with significant *q*-values for the comparison Arbo-AFI vs. Other-AFI were further evaluated by receiver operating characteristic (ROC) curve analysis, considering both individual predictors and their combination into panels comprising 3 markers. ROC analysis was performed using the PanelomiX R package (Robin et al., 2013). This approach identifies optimal combinations of biomarkers based on threshold-based rules under predefined performance constraints. For each model, the area under the curve (AUC) was calculated along with its 95% confidence interval (CI). Model performance was evaluated under a minimum accuracy constraint of 0.5, and corresponding sensitivity, specificity and Youden Index values were reported. DeLong’s test was used to statistically compare the AUCs of the individual markers and the combined panels against the baseline performance of CRP. A *p*-value < 0.05 was considered statistically significant. Statistical analyses were performed using R (version 4.3.3).

## 3 Results

The demographic characteristics of the studied population are reported in Supplementary Table S1. The cohort encompassed 89 patients with a laboratory-based diagnosis of arboviral infections (Supplementary Table S2) and 51 febrile patients with clinical or laboratory-based diagnosis of bacterial infections (*n* = 11, 21.6%), malaria (*n* = 21, 41.2%) or respiratory viral infections (*n* = 19, 37.2%). No differences in sex frequency, age distribution or body temperature were observed between the two groups. For *n* = 30 Arbo-AFI patients (34%) information about body temperature was not available, nonetheless they were included in the study since they were all presenting at the emergency department with symptoms.

**Figure 1:**
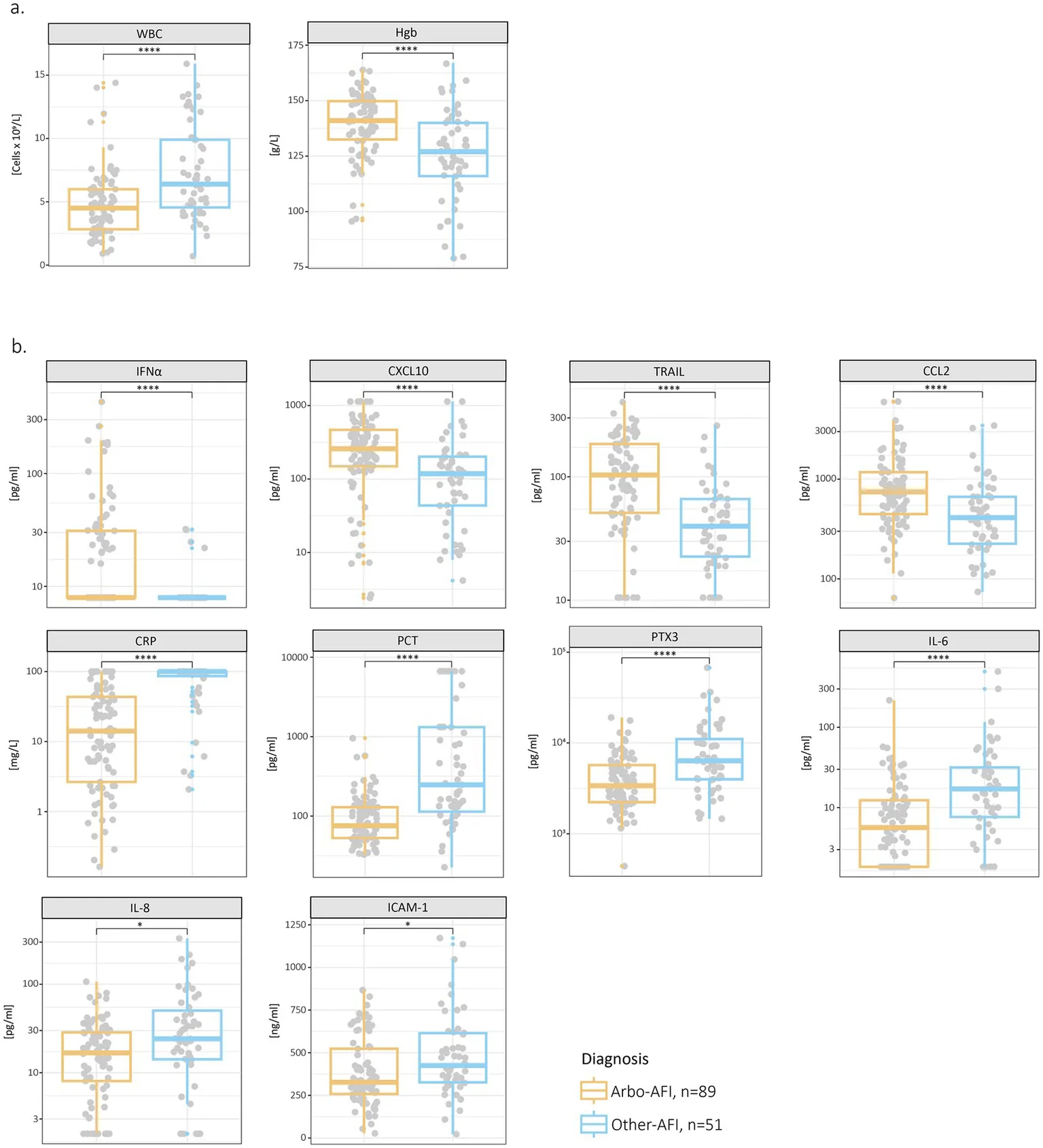
Haematological parameters **(a)** and soluble factors **(b)** significantly differentially abundant between patients with AFI of arboviral aetiology (Arbo-AFI) compared to those with other aetiology (Other-AFI). Statistical significance was computed with the Wilcoxon rank sum test followed by false discovery rate correction for multiple testing. Stars on the plots represent the *q*-value (i.e., FDR-adjusted *p*-value); ^*^ < 0.05; ^****^ ≤ 0.0001.

Among the 16 soluble immune mediators and the 4 haematological parameters, 12 were differentially abundant between Arbo-AFI and Other-AFI patients (Figure 1 and Supplementary Table S3). WBC count was significantly lower in Arbo-AFI compared to Other-AFI, with median values of 4.50 and 6.40 cells × 10^9^/L respectively, although below the threshold of 10 cells × 10^9^/L in both groups. Conversely, haemoglobin was raised in Arbo-AFI vs. Other-AFI, even though within its reference range and with a fold change of 1.11. Platelet and RBC counts were unaltered. Among soluble immune factors, IFNα, CXCL10, TRAIL and CCL2 were significantly higher in concentration in Arbo-AFI compared to Other-AFI (*q*-value ≤ 0.0001, FC 1.0–2.60). Acute phase reactants (CRP, PCT and PTX3), IL-6, IL-8 and ICAM-1 were instead more abundant in Other-AFI than in Arbo-AFI (*q*-value ≤ 0.014, FC 0.14–0.77). All immune factors that showed significant differences in the primary univariable comparisons, retained their statistical significance when further evaluated using multivariable logistic regression models adjusted for sample storage duration (data not shown). According to an exploratory statistical evaluation of the discriminatory ability of the different markers, CRP displayed the highest AUC (0.822), along with the best combination of sensitivity and specificity (0.883 and 0.760, respectively; Table 1). PCT and TRAIL showed AUC of 0.747 and 0.738, respectively, both not significantly different from the AUC of CRP (Table 1). To increase individual performances, marker combinations were considered. Three combinations (Comb 1: CRP + CXCL10 + IL-6; Comb 2: CRP + CXCL10 + PCT; Comb 3: Hgb + CRP + IL-6) differentiated between Arbo-AFI and Other-AFI with AUC > 0.9, all significantly higher compared to the AUC of each individual marker (*p* ≤ 0.015; Table 1).

**Table 1:** **(A)** ROC analysis for the markers showing significant different concentration in Arbo-AFI vs. Other-AFI. **(B)** Marker combinations in panels of 3 predictors.

Marker	AUC	95% CI	*p*-value^a^	SE	SP	Threshold	Youden^b^
a. Individual ROC							
CRP [mg/L]	0.822	0.752–0.891	1.000	0.883	0.760	<79.46	0.643
PCT [pg/ml]	0.747	0.668–0.825	0.093	0.753	0.740	<118.87	0.493
TRAIL [pg/ml]	0.738	0.664–0.813	0.067	0.636	0.840	>76.96	0.476
IL-6 [pg/ml]	0.719	0.638–0.800	0.017	0.818	0.620	<12.90	0.438
Hgb [g/L]	0.717	0.635–0.798	0.051	0.753	0.680	>133.50	0.433
CXCL10 [pg/ml]	0.694	0.612–0.777	0.034	0.688	0.700	>179.32	0.388
PTX3 [pg/ml]	0.692	0.613–0.771	0.004	0.584	0.800	<3,870.00	0.384
IFNα [pg/ml]	0.681	0.613–0.748	0.002	0.442	0.920	>11.91	0.362
WBC [cells*10^9^/L]	0.667	0.595–0.739	0.002	0.455	0.880	<3.85	0.335
CCL2 [pg/ml]	0.664	0.579–0.749	0.007	0.688	0.640	>508.76	0.328
ICAM-1 [ng/ml]	0.646	0.562–0.730	0.002	0.571	0.720	<340.33	0.291
IL-8 [pg/ml]	0.619	0.533–0.705	0.000	0.558	0.680	<17.00	0.238
b. Top 3 marker combinations							
CRP + CXCL10 + IL-6	0.915	0.865–0.965	0.005	0.870	0.900	CRP < 36.55	0.770
						CXCL10 > 224.34	
						IL-6 < 13.54	
CRP + CXCL10 + PCT	0.914	0.862–0.965	0.015	0.766	0.960	CRP < 31.15	0.789
						CXCL10 > 224.34	
						PCT < 283.88	
Hgb + CRP + IL-6	0.908	0.859–0.958	0.004	0.870	0.860	Hgb > 131.5	0.730
						CRP < 44.19	
						IL-6 < 9.86	

The top-3 combinations showing *p*-value < 0.05 compared to CRP—the best individual marker—and AUC > 0.9 are reported.

^a^Comparison with the best individual marker, i.e., CRP.

^b^Youden index: (SE + SP) − 1.

AUC, area under the ROC curve; CI, confidence interval; SE, sensitivity; SP, specificity.

**Figure 2:**
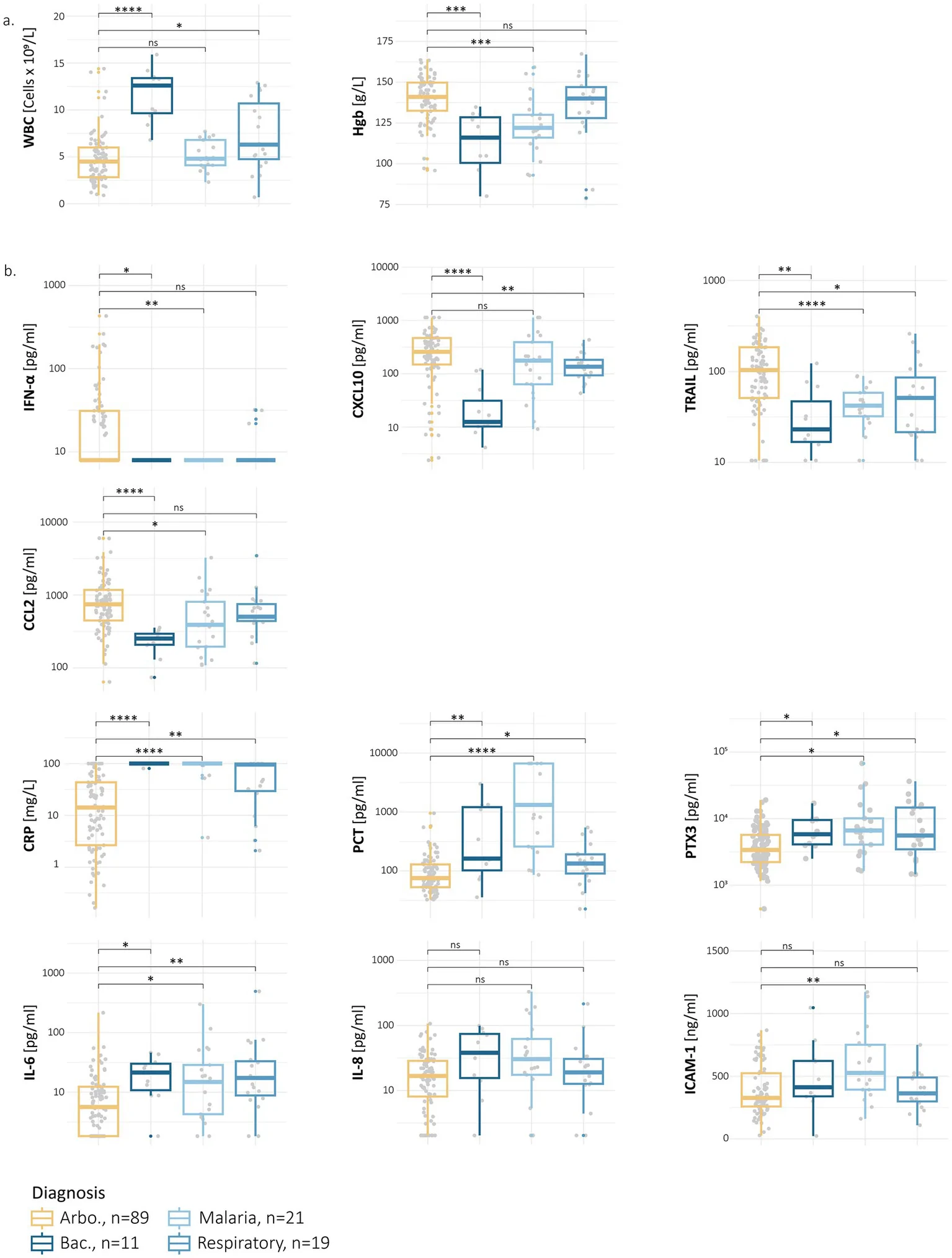
Haematological parameters **(a)** and soluble factors **(b)** significantly differentially abundant between patients with Arbo-AFI (Arbo.) compared with patients with AFI due to malaria, respiratory viruses or bacterial infections (Bac.) individually considered. Statistical significance was computed with the Wilcoxon rank sum test followed by false discovery rate correction for multiple testing. Stars on the plots represent the *q*-value (i.e., FDR-adjusted *p*-value); ^*^ < 0.05; ^**^ < 0.01; ^***^ < 0.001; ^****^ ≤ 0.0001; ns: non-significant.

Since Other-AFI was a heterogeneous group of infections, we compared the levels of the markers measured in Arbo-AFI to those observed in the different sub-categories (Figure 2, Supplementary Tables S4, S5). Considering markers having higher concentration in Arbo-AFI vs. Other-AFI, TRAIL was confirmed more abundant also when compared with all individual sub-groups, while CCL2 and IFNα were more abundant in Arbo-AFI vs. either malaria or bacterial infections, but not when compared with AFI sustained by respiratory viruses. Conversely, CXCL10 displayed the same concentration in Arbo-AFI and malaria, while it was significantly lower in respiratory bacterial infections and respiratory viruses. CRP, PCT, PTX3 and IL-6 were confirmed less abundant in Arbo-AFI also when compared with all sub-groups individually considered; while ICAM-1 was decreased in Arbo-AFI only when compared to malaria. IL-8 did not show any difference when Arbo-AFI were compared to the individual sub-groups of Other-AFI.

Haematological parameters showed variable profiles, with significantly raised WBC count in bacterial infections and respiratory viruses compared with Arbo-AFI, and higher Hgb in Arbo-AFI vs. either bacterial infections or malaria.

Although not differentially abundant between Arbo-AFI and Other-AFI overall, TNFα and PLT showed different profiles between Arbo-AFI and malaria, while IFNγ and RBC between Arbo-AFI and bacterial infections. Similarly, IL-10 was differentially abundant when compared with each individual subgroup but not when compared with the Other-AFI group as a whole, likely due to its high variability across the heterogeneous Other-AFI group (Supplementary Table S4).

**Figure 3:**
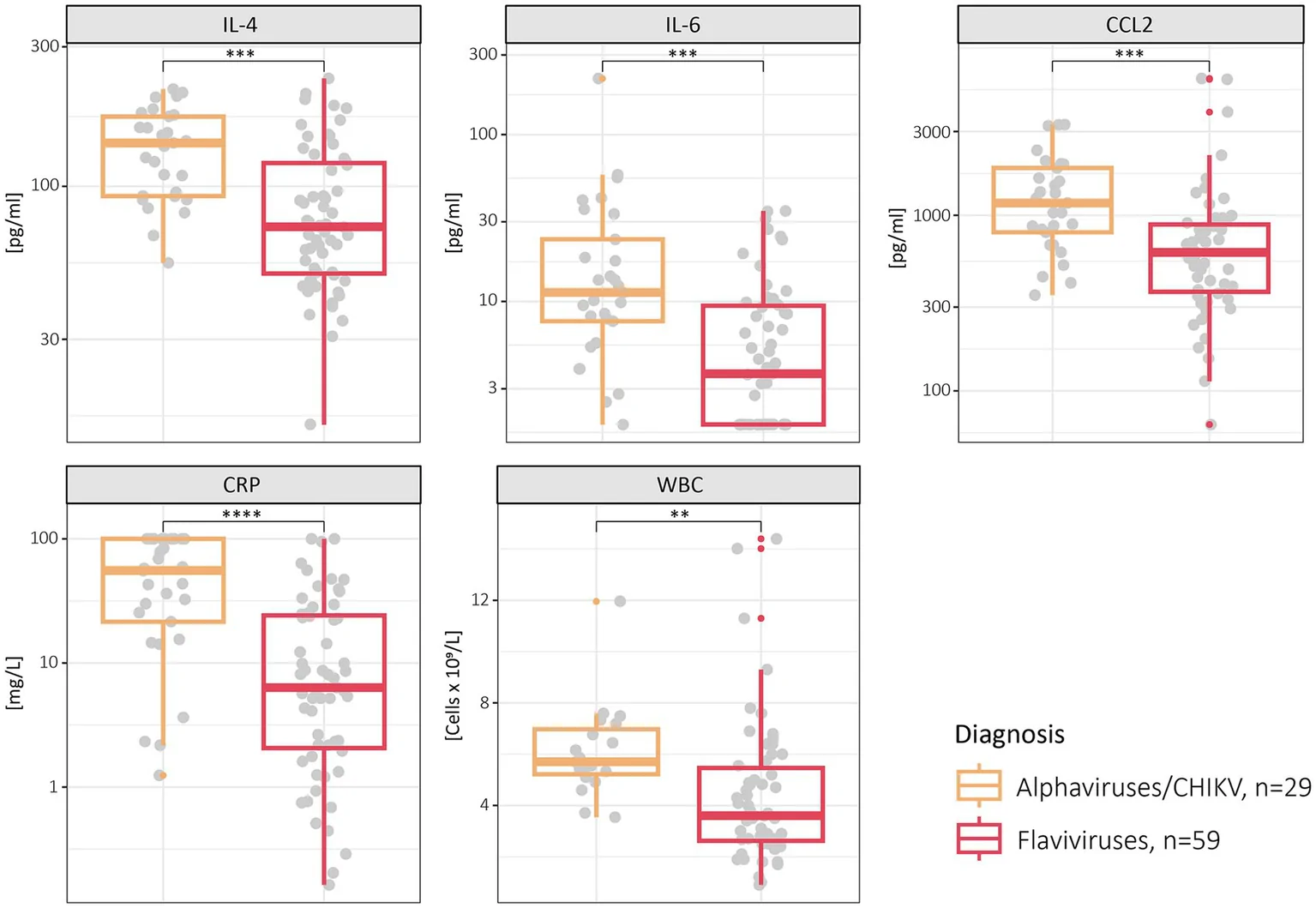
Haematological parameters and soluble factors significantly differentially abundant between patients with AFI due to alphaviruses (CHIKV) compared to AFI due to flaviviruses (DENV, WNV, TBEV, ZIKV). Statistical significance was computed with the Wilcoxon rank sum test followed by false discovery rate correction for multiple testing. Stars on the plots represent the *q*-value (i.e., FDR-adjusted *p*-value); ^**^ < 0.01; ^***^ < 0.001; ^****^ ≤ 0.0001.

The profile of the different markers was also evaluated within the Arbo-AFI group. Samples were grouped based on viral genus, i.e., *Alphavirus* (encompassing *n* = 29 CHIKV samples) or *Flavivirus* [*n* = 59, encompassing DENV (*n* = 45), WNV (*n* = 8), TBEV (*n* = 5) and ZIKV (*n* = 1)]. Alphaviruses, here represented only by CHIKV, displayed significantly higher concentration of IL-4, IL-6, CCL2 and CRP (*q*-value < 0.0006, FC ≥ 1.91), and higher WBC count (*q*-value = 0.0018, FC = 1.58) in comparison with flaviviruses (Figure 3 and Supplementary Table S6). Although CRP levels varied according to arboviral genus, its concentration in CHIKV infections was still significantly lower than in Other-AFI (Supplementary Table S6). Similarly, other markers showing promising discriminatory ability between Arbo- and Other-AFI—i.e., CXCL10, PCT and Hgb—showed significant different profiles also when alpha- or flavi-virus AFI are individually compared to Other-AFI. The levels of IL-6, instead, were not different between alphavirus-AFI and Other-AFI (Supplementary Table S6).

## 4 Discussion

In the present study we showed that AFI due to arboviral infections are accompanied by higher circulating IFNα, CXCL10, TRAIL and CCL2, and lower acute phase reactants (CRP, PCT and PTX3), acute innate cytokines (IL-6 and IL-8) and ICAM-1, in comparison with AFI due to other causes—including bacterial infections, malaria or respiratory viruses.

Prompt diagnosis of arboviral infections is essential for appropriate patient management and for the early detection of potential local outbreaks arising from imported cases. However, these infections typically present as an acute febrile illness accompanied by mild and largely non-specific symptoms (Forshey et al., 2010), complicating early clinical recognition. In this study, we built upon the established potential of host-derived biomarkers as fever triage tools (Leticia Fernandez-Carballo et al., 2021) to assess their utility for the early identification of arbovirus-associated AFI.

The host response to viral infections is driven by interferon-mediated and humoral immune responses, both of which are critical for achieving viral clearance from peripheral blood (Berber et al., 2024). In agreement with these pathophysiological features, in our study key regulators of the immune response to viruses—namely IFNα, CXCL10, TRAIL and CCL2 (Berber et al., 2024)—were significantly higher in Arbo-AFI compared with Other-AFI. The elevated levels of these markers in Arbo-AFI suggest early activation of dendritic cells, monocytes and macrophages, which are key components of innate immunity against viruses (Berber et al., 2024; Bartholomeeusen et al., 2023) and primary targets of several arboviruses (Bartholomeeusen et al., 2023; Her et al., 2010; Guzman et al., 2016). For instance, *in vitro* and *ex vivo* studies proposed that, during infection, monocytes may directly contribute to the increased release of IFNα and CXCL10 during CHIKV (Her et al., 2010), very likely as a strategy to control viral replication. Notably, CXCL10 and TRAIL remained significantly higher in arboviral cases even when strictly compared to respiratory viruses, supporting the strong association between these markers and acute arboviral infections. Evidence from studies comparing the host response across different arboviral infections in humans [e.g., (Tomalin et al., 2025; Dhenni et al., 2021)] and from our current understanding of the immunomodulation and immunopathology occurring during arboviral infections (Dash et al., 2024; da Silva et al., 2026; Lee et al., 2021) highlights that different arboviruses share some important features during the acute phase—such as increased expression of pro-inflammatory cytokines and activation of innate immune cells—while retaining virus-specific characteristics. Our results further support this evidence, as common alterations in immune mediators were observed when compared to Other-AFI, although with varying intensities (discussed later).

In our cohort, acute phase reactants (i.e., CRP, PTX3 and PCT) and acute innate cytokines (IL-6 and IL-8) were lower in Arbo-AFI compared to Other-AFI. Of these, all but IL-8, were confirmed lower in Arbo-AFI compared to either bacterial infections, respiratory viruses or malaria, when individually considered. The elevation of the acute phase response is a known mechanism of response to invading pathogens, and key molecules, namely CRP and PCT, are widely investigated as markers to differentiate viral and bacterial acute fevers both in high-income countries and in the tropics. Although variable results are reported by different studies, overall low levels of CRP and PCT are considered good predictors of viral acute fevers (Leticia Fernandez-Carballo et al., 2021; Lubell et al., 2015; Althaus et al., 2019; Wangrangsimakul et al., 2018), and they both showed some utility in detecting severe bacterial infections in persistent fever in the tropics (Van Duffel et al., 2022). Nonetheless, many caveats are still hampering their introduction into clinical practise, particularly in resource-limited settings, including the paucity of studies in sub-Saharan Africa (van Griensven et al., 2020). Our data showed that arboviral infections induced a lower activation of the acute phase reactants compared with other causes of AFI and, very interestingly, also compared with respiratory viruses. These observations suggest that low acute phase reactants might be important indicators of the arboviral origin of an acute fever. In our population, CRP was the marker showing the highest individual AUC (0.822), for differentiating between Arbo-AFI vs. Other-AFI. Importantly, its accuracy was significantly improved when combined with other markers, particularly with CXCL10/IL-6, CXCL10/PCT or Hgb/IL-6. (AUC > 0.90, *p*-value ≤ 0.015), even though the heterogeneity of the Other-AFI group might affect these estimates. It is however worth noticing that one of these combinations (i.e., CRP + CXCL10 + PCT) is very close to the panel for distinguishing bacterial and viral fevers which includes CRP, CXCL10 and TRAIL recently introduced on the market (Halabi et al., 2023). In our study, PCT seemed to outperform TRAIL in marker combinations, potentially due to the strong correlation of the latter with both CRP and CXCL10 (Supplementary Figure S1), suggesting that the contribution of TRAIL to the classification of Arbo-AFI patients might be marginal in comparison to CRP and CXCL10. The two other marker combinations showed equivalent ability to discriminate between the two groups of febrile patients. Of these markers, all but IL-6, displayed significantly different concentrations also when alpha- or flavivirus-AFI were individually compared to Other-AFI, to account for the variable inflammatory profile between the two types of viral genus. This observation supports the utility of these markers to distinguish Arbo-AFI from other causes of acute fever despite the heterogeneity of the former group. The utility of IL-6 might instead be limited since the observed results were affected by particularly low levels in flavivirus AFI. More in depth investigations of these markers are warranted to establish their accuracy in the early diagnosis of arboviral fever and to evaluate the robustness of the signatures across different arbovirus species.

An additional clinical challenge—particularly in endemic areas—is the differential diagnosis of arboviral infections, given their partly overlapping clinical presentations (Paniz-Mondolfi et al., 2016). A recent comparative analysis showed that different arboviral infections (ZIKV, DENV, CHIKV and Mayaro virus—MAYV) are accompanied by distinct cytokine profiles during the early febrile phase (Del Valle-Mendoza et al., 2026). Leveraging the relatively large number of *Alphavirus* (*n* = 29, here only represented by CHIKV) and *Flavivirus* (*n* = 59) cases in our cohort, we compared cytokine profiles according to arbovirus genus. Four markers (IL-4, IL-6, CCL2 and CRP) and WBC were higher in CHIKV compared to flaviviruses-infected samples. Interestingly, IL-4, IL-6 and CCL2 were all part of an immune signature of acute CHIKV identified by a meta-analysis (Teng et al., 2015). Our results suggest that, in our population, CHIKV might trigger a stronger pro-inflammatory environment compared to flaviviruses, potentially as a consequence of its short incubation and high viremia (Bartholomeeusen et al., 2023). Nonetheless, it has also been hypothesised that viral taxonomy might only marginally influence the host response, which instead appears to be virus specific, since an overall similar host response was reported in DENV and CHIKV paediatric patients, while a distinct mild innate response and a delayed adaptive response was revealed in ZIKV (Tomalin et al., 2025) or WNV (Fares-Gusmao et al., 2019). In our cohort, alterations observed between CHIKV and flaviviruses were confirmed when the comparison was restricted to CHIKV vs. DENV, although not surprisingly since the latter represented 76% of all flaviviruses analysed (data not shown).

Our study has some limitations. First, it was not possible to correlate the extent of the systemic response with viral load, since the latter parameter was not available, nor the correlation with body temperature as not available for some of the patients with arboviral infections, although they were all considered in the acute phase since presenting at the emergency department with symptoms. Similarly, data on the delay between symptom onset and sample collection were unavailable, preventing the assessment of a potential association between the immune profile and acute disease course. Indeed, the magnitude of the cytokine response has been shown to vary with both the timing since symptom onset and the type of infecting virus (Dhenni et al., 2021; Del Valle-Mendoza et al., 2026; Coutinho-da-Silva et al., 2022; Naveca et al., 2018) and, in the case of ZIKV, was accompanied by a bimodal viremia (Naveca et al., 2018). These observations indicate that accounting for the time since symptom onset when assessing circulating immune mediators may enable a more accurate evaluation of the pathophysiological processes during the acute phase of arboviral infections. However it must be reminded that, for clinical translation, arboviral fever triage markers should be independent of the time since symptom onset. Nonetheless, a prospective study should be performed to comprehensively establish the kinetics of the top-performing markers during disease progression and to assess the possible influence of the time delay between symptom onset and sample collection.

Our combinatorial analyses identified multiple promising marker combinations with AUC > 0.90 for distinguishing Arbo-AFI from Other-AFI, including the signature encompassing CRP, PCT and CXCL10. These observations might have been partly affected by the retrospective design of our study and the heterogeneity of the Other-AFI group, which encompassed clinically different febrile syndromes. To overcome this limitation, a prospective clinical validation of the best-performing markers will be required to evaluate their potential as novel triage tests for arboviral fever more comprehensively. A larger validation cohort will enable also to define the clinical relevance of the marker signatures regarding specific causes of non-arboviral AFI.

In summary, we showed that AFI due to arboviral infections are accompanied by increased mediators of the anti-viral responses and decreased acute phase reactants and acute innate cytokines, when compared to other causes of AFI including other viral conditions. Moreover, some of the best performing markers—including CXCL10, TRAIL, CRP and PCT—showed distinct profiles in Arbo-AFI and AFI due to respiratory viruses. Our exploratory investigations highlighted signatures encompassing CRP, PCT, CXCL10, IL-6 or Hgb as able to detect arboviral fever with AUC ≥ 0.91, paving the way for more in depth clinical validation of host immune mediators as arboviral fever triage tests.

## Data Availability

The de-identified dataset, containing demographic information and experimental raw data, is accessible via the Zenodo repository (DOI: 10.5281/zenodo.22009678).
