# Supplementary material for: Host-derived immune signatures as biomarkers to differentiate arboviral acute febrile illness from other causes of acute fever
Source: Front Microbiol. 2026 Sep 3;17:1926383. doi: 10.3389/fmicb.2026.1926383 (PMC13582234; doi:10.3389/fmicb.2026.1926383)

Supplementary material

Supplementary Table S1. Demographic description of the analysed population.

	**Arbo-AFI (n=89)**	**Other-AFI (n=51)**	**p-value[a]**
Age (years), median [range]	49 [19 - 87]	55 [22 - 96]	ns
Sex (F), n (%)	39 (43.8%)	16 (31.4%)	ns
Max body temperature (°C), median [range][b]	38.6 [38.0 - 41.0]	38.5 [38.0 - 40.2]	ns
Sub-groups, n (%)			
DENV	45 (50.6%)		
WNV	8 (9.0%)		
CHIKV	29 (32.6%)		
TBEV	5 (5.6%)		
ZIKV	1 (1.1%)		
OROV	1 (1.1%)		
Bacterial infections[c]		11 (21.6%)	
Malaria[d]		21 (41.2%)	
Sars-CoV-2		17 (33.3%)	
Influenza A virus		2 (3.9%)	
Delay[e], number of days (%)			
0	87 (97.8%)	28 (54.9%)	
1	2 (2.2%)	19 (37.3%)	
≥2	0	4 (7.8%)	

[a] Mann-Whitney test for comparison between continuous variables; Fisher's exact test for categorical variables
[b] unknown data about fever for n=30 Arbo-AFI patients, all admitted to the emergency department
[c] The group included patients with radiological signs of bacterial pneumonia with or without a positive pneumococcal urinary antigen test; documented abdominal, cutaneous or pelvic abscesses with or without bacteraemia confirmed by positive blood culture; microbiologically confirmed bacteraemia originating from documented urinary tract infection
[d] *P. falciparum* n=15; *P. malariae* n=1; *P. ovale* n=1; Mixed forms n=2; *Plasmodium* spp. n=2
[e] Delay between patient's admission/diagnosis and collection of the serum sample analysed in the present study

Supplementary Table S2. Proportion of arboviral fever cases confirmed by each diagnostic method. Each patient was positive to at least one of the reported tests.

	DENV (n=45)	CHIKV (n=29)	WNV (n=8)	TBEV (n=5)	ZIKV (n=1)	OROV (n=1)
PCR in blood, n (%)						
Positive	41 (91.1%)	29 (100%)	7 (87.5%)	2 (40%)	1 (100%)	1 (100%)
Negative	2 (4.4%)	0	1 (12.5%)	0	0	0
nd	2(4.4%)	0	0	3 (60%)	0	0
PCR in urine, n (%)						
Positive	35 (77.8%)	12 (41.4%)	7 (87.5%)	-	1 (100%)	1 (100%)
Negative	7 (15.5%)	15 (51.7%)	1 (12.5%)	-	0	0
nd	3 (6.7%)	2 (6.9%)	0	5 (100%)	0	0
NS1 antigen, n (%)[a]						
Positive	38 (84.4%)	na	na	na	na	na
Negative	6 (13.3%)	na	na	na	na	na
nd	1 (2.2%)	na	na	na	na	na
IgM in CSF, n (%)[b]						
Positive	na	na	na	3 (60%)	na	na
Negative	na	na	na	0	na	na
nd	na	na	na	2 (40%)	na	na

[a]NS1 antigenic test only performed for DENV; [b]IgM serology in CSF only performed for TBEV
PCR: polymerase chain reaction; CSF: cerebrospinal fluid; na: not applicable; nd: not done.

Supplementary Table S3. Concentration of the assessed markers in patients with arboviral AFI (Arbo-AFI) or AFI of other aetiology (Other-AFI)

		**Arbo-AFI**			**Other-AFI**			**Arbo-AFI/Other-AFI**		
	**Reference range**	**n**	**Median**	**Range**	**n**	**Median**	**Range**	**FC**	**p-value[a]**	**q-value[b]**
**Haematology**										
**WBC [cells x 10^9/L]**	5.2 - 12.4	78	4.50	0.90 - 14.40	51	6.40	0.70 - 15.90	**0.70**	<0.0001	**0.0001**
RBC [cells x 10^{12}/L]	4.2 - 5.4	78	4.71	2.60 - 6.20	51	4.56	2.11 - 5.64	1.03	0.0388	0.0596
**Hgb [g/L]**	120 - 160	78	141.00	96.00 - 164.00	51	127.00	79.00 - 167.00	**1.11**	<0.0001	**0.0001**
PLT [cells x 10^9/L]	130 - 400	78	169.50	34.00 - 482.00	51	164.00	24.00 - 750.00	1.03	0.3600	0.4500
**Immune factors**										
**CRP [mg/L]**	--	89	14.15	0.16 - 100.00	51	100.00	2.08 - 100.00	**0.14**	<0.0001	**<0.0001**
**PTX3 [pg/ml]**	--	89	3382.00	440.0 - 19051.0	51	6364.00	1459.00 – 67404.00	**0.53**	<0.0001	**0.0001**
**CCL2 [pg/ml]**	--	89	745.89	64.30 - 6011.98	51	410.96	74.11 - 3452.06	**1.81**	<0.0001	**0.0001**
**CXCL10 [pg/ml]**	--	89	258.40	2.40 - 1135.01	51	117.96	4.16 - 1135.01	**2.19**	<0.0001	**0.0001**
**ICAM-1 [ng/ml]**	--	89	327.03	27.59 - 867.14	50	424.95	22.54 - 1172.17	**0.77**	0.0081	**0.0135**
**IFNα [pg/ml]**	--	88	7.94	7.94 - 430.99	51	7.94	7.94 - 32.00	**1.00**	<0.0001	**<0.0001**
IFNγ [pg/ml]	--	89	18.58	3.64 - 1430.54	51	23.56	3.64 - 953.69	0.79	0.9221	0.9221
IL-2 [pg/ml]	--	89	10.88	10.88 - 106.18	51	10.88	10.88 – 121.94	1.00	0.9094	0.9221
IL-4 [pg/ml]	--	89	90.02	15.33 - 233.76	51	76.44	15.33 – 175.22	1.18	0.2022	0.2696
**IL-6 [pg/ml]**	--	89	5.64	1.83 - 216.68	51	17.10	1.83 – 495.33	**0.33**	<0.0001	**0.0001**
**IL-8 [pg/ml]**	--	89	16.76	2.04 - 106.87	51	24.21	2.04 – 330.37	**0.69**	0.0074	**0.0134**
IL-10 [pg/ml]	--	89	19.15	1.94 - 334.22	51	13.69	1.94 - 1777.18	1.40	0.4145	0.4877
**PCT [pg/ml]**	--	89	75.46	32.57 - 956.17	51	247.60	22.63 - 6659.98	**0.30**	<0.0001	**<0.0001**
TNFα [pg/ml]	--	89	13.71	1.47 - 34.57	51	14.86	2.80 – 116.17	0.92	0.0713	0.1019
**TRAIL [pg/ml]**	--	89	103.47	10.50 - 403.11	51	39.77	10.50 - 259.92	**2.60**	<0.0001	**<0.0001**
VCAM-1 [ng/ml]	--	89	3089.04	279.44 - 4814.14	51	3253.36	774.64 - 4814.14	0.95	0.5149	0.5721

[a] Wilcoxon rank sum test
[b] False discovery rate correction for multiple testing
FC: fold change

Supplementary Table S4. Concentration of the assessed markers in arboviral AFI (Arbo-AFI) vs. other causes of AFI individually considered.

	Arbovirsues		Bacterial infections		Malaria		Respiratory viruses		Multiple comparisons[b]		
	**n**	**Median [range]**	**n**	**Median [range]**	**n**	**Median [range]**	**n**	**Median [range]**	**Arbo. vs. Bac.**	**Arbo. vs. Mal.**	**Arbo. vs. Resp.**
**Haematology**											
WBC [cells x 10^9/L]	78	4.50 [0.90 - 14.40]	11	12.60 [6.80 - 15.90]	21	4.80 [2.30 - 7.70]	19	6.30 [0.70 - 12.90]	<0.0001	0.1860	0.0104
RBC [cells x 10^{12}/L]	78	4.71 [2.60 - 6.20]	11	4.12 [2.88 - 5.18]	21	4.36 [3.02 - 5.64]	19	4.69 [2.11 - 5.30]	0.0058	0.0631	0.5186
Hgb [g/L]	78	141.00 [96.00 - 164.00]	11	116.00 [80.00 - 135.00]	21	122.00 [93.00 - 159.00]	19	140.00 [79.00 - 167.00]	0.0002	0.0002	0.5244
PLT [cells x 10^9/L]	78	169.50 [34.00 - 482.00]	11	235.00 [130.00 - 698.00]	21	84.00 [24.00 - 219.00]	19	200.00 [90.00 - 750.00]	0.0286	<0.0001	0.2038
**Immune factors**											
CRP [mg/L]	89	14.15 [0.16 - 100.00]	11	100.00 [80.51 - 100.00]	21	100.00 [3.72 - 100.00]	19	95.61 [2.08 - 100.00]	<0.0001	<0.0001	0.0019
PTX3 [pg/ml]	89	3382.00 [440.00 - 19051.0]	11	5824.00 [3976.00 - 9799.00]	21	6636.00 [1638.00 - 67404.00]	19	5572.00 [1459.00 - 36401.00]	0.0226	0.0152	0.0226
CCL2 [pg/ml]	89	745.89 [64.30 - 6011.98]	11	253.03 [74.11 – 356.17]	21	390.55 [109.10 - 3247.16]	19	503.85 [115.79 - 3452.06]	<0.0001	0.0203	0.1028
CXCL10 [pg/ml]	89	258.40 [2.40 - 1135.01]	11	12.54 [4.16 – 119.46]	21	176.80 [9.22 - 1135.01]	19	136.05 [43.19 - 433.17]	<0.0001	0.1755	0.0048
ICAM-1 [ng/ml]	89	327.03 [27.59 - 867.14]	11	411.48 [22.54 – 1047.47]	20	526.51 [160.23 - 1172.17]	19	362.98 [108.59 - 750.14]	0.2514	0.0088	0.6456
IFNα [pg/ml]	88	7.94 [7.94 - 430.99]	11	7.94 [7.94 - 7.94]	21	7.94 [7.94 - 7.94]	19	7.94 [7.94 - 32.00]	0.0172	0.0012	0.0563
IFNγ [pg/ml]	89	18.58 [3.64 - 1430.54]	11	3.64 [3.64 - 318.53]	21	23.56 [3.64 - 953.69]	19	47.14 [3.64 - 379.87]	0.0090	0.7575	0.1562
IL-2 [pg/ml]	89	10.88 [10.88 - 106.18]	11	10.88 [10.88 – 81.50]	21	20.71 [10.88 - 121.94]	19	10.88 [10.88 - 24.97]	0.6128	0.2238	0.3149
IL-4 [pg/ml]	89	90.02 [15.33 - 233.76]	11	70.67 [30.67 – 175.22]	21	98.97 [15.33 - 152.96]	19	73.31 [37.84 - 149.71]	0.3321	0.9182	0.3321
IL-6 [pg/ml]	89	5.64 [1.83 - 216.68]	11	21.42 [1.83 – 46.24]	21	14.87 [1.83 - 300.96]	19	17.38 [1.83 - 495.33]	0.0128	0.0241	0.0076
IL-8 [pg/ml]	89	16.76 [2.04 - 106.87]	11	38.03 [2.04 - 1 – 98.93]	21	30.33 [2.04 - 330.37]	19	19.02 [2.04 - 215.34]	0.0920	0.0920	0.4811
IL-10 [pg/ml]	89	19.15 [1.94 - 334.22]	11	1.94 [1.94 – 10.55]	21	40.64 [1.94 - 1777.18]	19	9.49 [1.94 - 29.19]	0.0004	0.0008	0.0167
PCT [pg/ml]	89	75.46 [32.57 - 956.17]	11	162.03 [35.77 – 3022.87]	21	1317.18 [85.82 - 6659.98]	19	133.55 [22.63 - 545.18]	0.0073	<0.0001	0.0173
TNFα [pg/ml]	89	13.71 [1.47 - 34.57]	11	11.34 [4.35 – 37.15]	21	26.58 [9.97 - 116.17]	19	13.41 [2.80 - 50.28]	0.5539	0.0001	0.6169

TRAIL [pg/ml]	89	103.47 [10.50 - 403.11]	11	23.08 [10.50 - 122.60]	21	42.12 [10.50 - 89.17]	19	51.16 [10.50 - 259.92]	0.0014	0.0001	0.0121
VCAM-1 [ng/ml]	89	3089.04 [279.44 - 4814.14]	11	1916.06 [774.64 - 4814.14]	21	4814.14 [972.49 - 4814.14]	19	3315.19 [1924.01 - 4814.14]	0.3471	0.3471	0.4617

[a] The group "Meningitis/Encephalitis was not included in statistical comparisons due to the limited sample size

[b] Wilcoxon test with FDR correction

Arbo.: arbovirus; Bac.: Bacterial infections; Mal.: Malaria; Resp.: respiratory viruses

Supplementary Table S5. ROC analysis for Arbo-AFI vs. other-AFI subgroups individually considered.

	Arbo (n=89) vs. Mal. (n=21)						Arbo (n=89) vs. Bac. (n=11)						Arbo (n=89) vs. Resp. (n=19)					
**Marker**	**AUC**	**95% CI**	**SE**	**SP**	**Threshold**	**Youden[a]**	**AUC**	**95% CI**	**SE**	**SP**	**Threshold**	**Youden[a]**	**AUC**	**95% CI**	**SE**	**SP**	**Threshold**	**Youden[a]**
WBC [cells x 10^9/L]	0.595	0.473-0.716	0.45	0.86	3.85	0.31	0.955	0.912-0.998	0.94	0.91	8.10	0.85	0.701	0.560-0.842	0.94	0.42	8.00	0.36
Hgb [g/L]	0.783	0.655-0.911	0.74	0.81	133.50	0.55	0.893	0.816-0.971	0.68	1.00	135.50	0.68	0.548	0.392-0.703	0.33	0.79	148.50	0.12
CRP [mg/L]	0.889	0.814-0.964	0.78	0.95	49.86	0.73	0.931	0.890-0.971	0.85	1.00	79.46	0.85	0.742	0.617-0.866	0.63	0.79	26.15	0.42
PTX3 [pg/ml]	0.713	0.583-0.842	0.78	0.62	6123.50	0.4	0.735	0.598-0.873	0.57	0.91	3870.00	0.48	0.686	0.534-0.838	0.93	0.42	9378.50	0.35
CCL2 [pg/ml]	0.681	0.540-0.823	0.76	0.57	438.63	0.33	0.922	0.869-0.975	0.83	1.00	357.26	0.83	0.626	0.498-0.755	0.56	0.74	683.43	0.3
CXCL10 [pg/ml]	0.602	0.455-0.750	0.56	0.71	224.34	0.27	0.916	0.861-0.972	0.83	1.00	120.85	0.83	0.716	0.610-0.822	0.70	0.74	174.51	0.44
ICAM-1 [ng/ml]	0.729	0.606-0.852	0.57	0.85	351.87	0.42	0.642	0.466-0.819	0.54	0.82	337.01	0.36	0.534	0.401-0.667	0.44	0.74	307.38	0.18
IFNα [pg/ml]	0.722	0.669-0.774	1.00	0.44	11.9	0.44	0.722	0.669-0.774	1.00	0.44	11.9	0.44	0.631	0.531-0.731	1.00	0.24	32.6	0.24
IL-6 [pg/ml]	0.675	0.542-0.808	0.80	0.57	14.36	0.37	0.763	0.613-0.912	0.62	0.91	8.69	0.53	0.734	0.608-0.861	0.67	0.74	9.86	0.41
IL-8 [pg/ml]	0.67	0.522-0.818	0.83	0.48	34.57	0.31	0.701	0.496-0.905	0.80	0.64	31.18	0.44	0.562	0.419-0.705	0.36	0.84	12.04	0.2
PCT [pg/ml]	0.927	0.861-0.992	0.91	0.86	207.31	0.77	0.770	0.588-0.952	0.75	0.73	130.13	0.48	0.685	0.546-0.825	0.73	0.63	119.97	0.36
TRAIL [pg/ml]	0.801	0.720-0.883	0.56	1.00	90.65	0.56	0.824	0.706-0.942	0.90	0.73	32.98	0.63	0.701	0.565-0.838	0.65	0.74	72.74	0.39

Arbo.: arbovirus; Bac.: Bacterial infections; Mal.: Malaria; Resp.: respiratory viruses. [a]Youden index: (SE+SP)-1
AUC: area under the ROC curve; CI: confidence interval; SE: sensitivity; SP: specificity.
Only markers showing significantly different concertation in the main comparison Arbo-AFI vs. Other-AFI are reported.

Supplementary Table S6. Comparison of marker concentration in patients with acute fever due to alphaviruses/CHIKV, flaviviruses or non-arboviral causes.

	**Alphaviruses/CHIKV[a]**			**Flaviviruses**			**Other-AFI**			**Multiple comparisons[b]**		
	**n**	**Median**	**Range**	**n**	**Median**	**Range**	**n**	**Median**	**Range**	**CHIKV vs. Falvi**	**CHIKV vs. Other-AFI**	**Flavi vs. Other-AFI**
**Haematology**												
WBC [cells x 10^9/L]	19	5.70	3.54 - 11.96	58	3.60	0.90 - 14.40	51	6.40	0.70 - 15.90	**0.001**	0.513	**<0.0001**
RBC [cells x 10^{12}/L]	29	4.72	3.97 - 5.42	58	4.72	2.60 - 6.20	51	4.56	2.11 - 5.64	0.583	0.360	0.113
Hgb [g/L]	29	145.00	103.00 - 163.00	58	141.00	96.00 - 164.00	51	127.00	79.00 - 167.00	0.710	**0.003**	**<0.0001**
PLT [cells x 10^9/L]	29	186.00	104.00 - 258.00	58	162.50	34.00 - 482.00	51	164.00	24.00 - 750.00	0.209	0.209	0.671
**Immune factors**												
CRP [mg/L]	29	55.59	1.24 - 100.00	59	6.33	0.16 - 100.00	51	100.00	2.08 - 100.00	**<0.0001**	**0.001**	**<0.0001**
PTX3 [pg/ml]	29	3560.00	1327.00 - 19051.00	59	3337.00	440.00 - 17635.00	51	6364.00	1459.00 – 67404.00	1.000	**0.002**	**0.001**
CCL2 [pg/ml]	29	1175.32	351.31 - 3290.91	59	614.81	64.30 - 6011.98	51	410.96	74.11 - 3452.06	**<0.0001**	**<0.0001**	**0.008**
CXCL10 [pg/ml]	29	337.04	24.52 - 1135.01	59	211.40	2.40 - 1135.01	51	117.96	4.16 - 1135.01	**0.033**	**<0.0001**	**0.006**
ICAM-1 [ng/ml]	29	354.79	128.75 - 829.37	59	327.03	27.59 - 867.14	50	424.95	22.54 - 1172.17	0.338	0.212	**0.020**
IFNα [pg/ml]	29	7.94	7.94 - 104.01	58	7.94	7.94 - 430.99	51	7.94	7.94 - 32.00	0.976	**<0.0001**	**<0.0001**
IFNγ [pg/ml]	29	28.50	3.64 - 192.93	59	17.14	3.64 - 1430.54	51	23.56	3.64 - 953.69	0.772	0.772	0.772
IL-2 [pg/ml]	29	10.88	10.88 - 33.05	59	10.88	10.88 - 106.18	51	10.88	10.88 – 121.94	0.983	0.983	0.983
IL-4 [pg/ml]	29	140.61	54.81 - 215.22	59	72.75	15.33 - 233.76	51	76.44	15.33 – 175.22	**<0.0001**	**<0.0001**	0.493
IL-6 [pg/ml]	29	11.32	1.83 - 216.68	59	3.69	1.83 - 34.85	51	17.10	1.83 – 495.33	**<0.0001**	0.324	**<0.0001**
IL-8 [pg/ml]	29	21.96	2.04 - 106.87	59	14.94	2.04 - 79.65	51	24.21	2.04 – 330.37	0.058	0.336	**0.007**
IL-10 [pg/ml]	29	23.14	1.94 - 93.86	59	10.80	1.94 - 334.22	51	13.69	1.94 - 1777.18	0.212	0.146	0.966
PCT [pg/ml]	29	109.95	34.57 - 956.17	59	66.81	32.57 - 309.24	51	247.60	22.63 - 6659.98	**0.015**	**0.001**	**<0.0001**
TNFa [pg/ml]	29	16.48	4.89 - 30.29	59	12.74	1.47 - 34.57	51	14.86	2.80 – 116.17	0.218	0.568	0.113
TRAIL [pg/ml]	29	120.41	26.14 - 253.26	59	85.28	10.50 - 403.11	51	39.77	10.50 - 259.92	0.051	**<0.0001**	**<0.0001**
VCAM-1 [ng/ml]	29	2855.11	279.44 - 4814.14	59	3150.51	377.39 - 4814.14	51	3253.36	774.64 - 4814.14	0.282	0.282	0.920

[a] In our cohort alphaviruses were represented only by CHIKV
[b] Wilcoxon test with FDR correction
Flavi.: flaviviruses.

Figure S1. Correlation matrix of the concentration of soluble markers and haematological parameters assessed. Colour scale indicates the Spearman rho coefficient, grey dots indicate significant correlations.

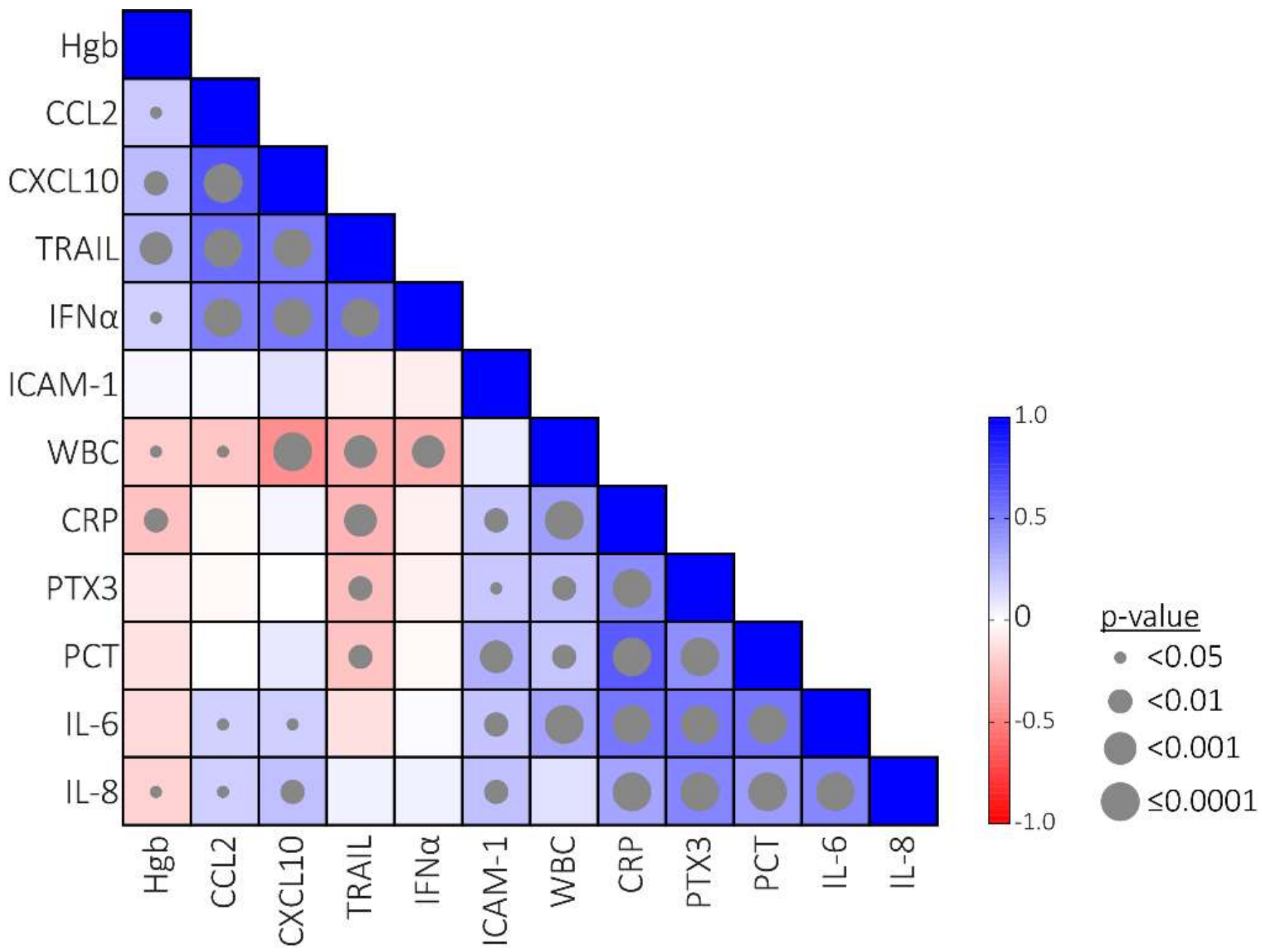

Supplement: Supplementary file 1 [file Data_Sheet_1.PDF]
